# Chronic idiopathic urinary retention and Fowler's syndrome in women: A multidisciplinary framework for assessment and nonsurgical management

**DOI:** 10.1002/bco2.70240

**Published:** 2026-07-02

**Authors:** Sarah McRae, Caoimhe McLoughlin, Carolyn Davie, Claire Hentzen, Dani Coombe, Claire Todd, Clare Nicholson, Beatrice Garcin, Glenn Nielsen, Helen Simpson, Laura McWhirter, Helen Jinadu, Jalesh Panicker, Anne Sanderson, Alan Carson, Martine Cornillet, Miriam Gamble, Voula Granitsiosis, Sioned Hancock, Collete Haslam, Leila Heelas, Victoria Hulman, Emily Jay, Mahreen Pakzad, Caroline Selai, Natalia Vasquez, Louise Young, Jon Stone, Ingrid Hoeritzauer

**Affiliations:** ^1^ NHS Lothian Edinburgh UK; ^2^ University of Edinburgh Edinburgh UK; ^3^ NHS Tayside Dundee UK; ^4^ Hôpital Universitaire Pitié Salpêtrière Paris France; ^5^ Fowler's Syndrome UK London UK; ^6^ King's College London London UK; ^7^ University College London London UK; ^8^ Hopital Avicenne Bobigny France; ^9^ St George's Hospital London London UK; ^10^ NHS Fife Fife UK; ^11^ Department of Neuropsychiatry South London and Maudsley NHS Foundation Trust London UK; ^12^ Department of Uro‐neurology University College London London UK; ^13^ Women's Health Dublin Dublin Ireland; ^14^ Physiotherapy Research Unit Oxford University Hospitals NHS Foundation Trust Oxford UK; ^15^ Neurotherapy Services Edinburgh Edinburgh UK

**Keywords:** Fowler's syndrome, chronic idiopathic urinary retention, occupational therapy, pain, physiotherapy, psychology, urinary retention

## Abstract

Chronic idiopathic urinary retention and Fowler's syndrome are a debilitating problem primarily affecting women, characterised by an inability to urinate in the absence of any identifiable structural or other cause. An increasingly wide range of multifactorial predisposing, precipitating, and perpetuating biopsychosocial factors are being recognised. This has led to interest in nonsurgical treatments that may complement existing surgical approaches. We reviewed existing literature on nonsurgical management and combined this information with collective multidisciplinary professional and lived experience, to produce a new definition of chronic idiopathic urinary retention (the Fife Definition) and a framework for the assessment and nonsurgical treatment for women with chronic idiopathic urinary retention. The article provides a stepped care approach to nonsurgical treatment including explanation and formulation, basic bladder healthcare, optimising catheterisation, management of relevant comorbidities, multidisciplinary treatment and additional treatment options and emerging therapies.

## 1 INTRODUCTION

Chronic idiopathic urinary retention is a debilitating problem primarily affecting women, characterised by an inability to urinate in the absence of any identifiable structural or other cause.^1^ Fowler's Syndrome describes a subset of women with chronic idiopathic urinary retention with demonstrable overactivity of the external urethral sphincter causing urinary retention.^2^ Chronic idiopathic urinary retention/Fowler's syndrome continues to be described using various terms including external urethral sphincter overactivity, hypertonic non‐relaxing urethral sphincter and nonneurogenic neurogenic bladder. We are referring to these various conditions but have chosen chronic idiopathic urinary retention for ease and clarity in this article. The precise incidence of chronic idiopathic urinary retention is unclear, but Fowler's syndrome is thought to affect 0.2 women per 100 000 per year.

### 1.1 Aims and challenges

In recent years, research on common comorbidities, especially chronic primary pain and functional neurological disorder, has led to reappraisal of chronic idiopathic urinary retention and Fowler's syndrome. Multifactorial biopsychosocial predisposing, precipitating and perpetuating factors are increasingly being recognised. This has led to interest in nonsurgical treatments that may complement existing surgical approaches.

There has been a lack of research into rehabilitative and nonsurgical treatments generally in chronic idiopathic urinary retention, with an absence of randomised controlled studies of physiotherapy or psychotherapy. The existing literature also contains little guidance for those seeking practical nonsurgical treatment options. Part of the challenge in this field arises from the numerous terms used to describe chronic idiopathic urinary retention and Fowler's syndrome.

We aimed to reconcile the different terms, drawing together existing literature on nonsurgical management and combine this information with our collective multidisciplinary professional and lived experience, to produce a preliminary consensus framework for the assessment and nonsurgical treatment for women with chronic idiopathic urinary retention.

We describe these as a *multidisciplinary framework* in recognition that many of the ideas are based on lived experience, expert knowledge and clinical practice/exposure as well as rehabilitative principles used in other urological and related conditions. We aimed to produce this framework as a springboard for further studies and treatment trials. Dysfunctional voiding occurs in men and women, although it is more common in women. This article is focused on the assessment and treatment of women as that is where the expertise of the authorship lay. These assessments and treatments are likely to be helpful for male patients as well, but further study is needed.

## 2 REVIEW OF EXISTING LITERATURE

We reviewed the existing literature on all surgical and nonsurgical treatment for chronic idiopathic urinary retention. Our recent systematic review found 52 papers (Mason et al. in submission), (*n* = 1471 patients, majority female). Despite chronic idiopathic urinary retention and Fowler's syndrome being seen by many as a predominantly UK urological issue, 21% were from the United States, and 17% were from the United Kingdom; most were retrospective cohort studies. Most studies involved patients diagnosed with voiding dysfunction. Pharmacological treatments were most common (botulinum toxin in eight studies), followed by surgery and then sacral neuromodulation. There were six studies of physiotherapy (*n* = 254 patients) and seven of psychotherapy (only *n* = 15 patients). Outcome measures were heterogeneous, making comparison of results difficult. We found no randomised clinical trials that included nonsurgical treatments. We drew on this evidence to inform our preliminary consensus framework, in addition to other sources.

We also collaborated with Fowler's Syndrome UK www.fowlerssyndrome.co.uk, the only patient organisation for chronic idiopathic urinary retention, to survey 265 patients, examining a wide range of experiences with their condition and its treatment. This showed that nonsurgical approaches were being offered to at least 25% of patients (e.g., cognitive behavioural and other psychological therapy, tibial nerve stimulation and physiotherapy).^3^

Finally, we carried out a survey of physiotherapists with a special interest in Pelvic, Obstetric and Gynaecological Physiotherapy and neurophysiotherapists in the United Kingdom. The results of this survey of 100 health professionals showed that there was considerable interest, although little experience in nonsurgical treatment. Treatments used by respondents included education, pelvic floor physiotherapy, biofeedback and tibial nerve stimulation but with negative perceptions about the potential for improvement.^4^

## 3 DEVELOPMENT OF THE FRAMEWORK

Twenty‐two participants with experience of treatment of chronic idiopathic urinary retention were invited to a 2‐day meeting (7–8 September 2023) in Fife, Scotland, and included neurologists, urologists, a uro‐neurologist, a psychiatrist, neurophysiotherapists, pelvic health physiotherapists, occupational therapists, bladder specialist nurses, a clinical psychologist and patient representatives who were from the United Kingdom and France. Eight participants, who were identified as being experts in the field, were unable to attend in person but were invited to contribute to the document.

During the meeting, findings from the literature review, patient survey and physiotherapy survey were presented. This was followed by a presentation of a case series of patients with good outcomes, and a presentation of lived experience of chronic idiopathic urinary retention by one of the coauthors. An iterative process of discussion followed, which was facilitated by one of the authors (IH).

## 4 TERMINOLOGY—A DEFINITION OF CHRONIC IDIOPATHIC URINARY RETENTION

The group recognised that the field was hampered by multiple synonymous terms and a lack of standard definitions of chronic idiopathic urinary retention. Our starting point was current definitions relevant to urinary retention from the International Urogynaecological Association (IUGA) and International Continence Society^5^

- *Urinary retention*: Complaint of the inability to pass urine despite persistent effort.
- *Acute urinary retention*: This is defined as a generally (but not always) painful, palpable or percussable bladder when the patient is unable to pass any urine when the bladder is full.
- *Chronic retention of urine*: a nonpainful bladder, where there is a chronically high post‐void residual.
- *Bladder outflow obstruction*: This is the generic term for obstruction during voiding. It is a reduced urine flow rate and/or presence of a raised post‐void residual and an increased detrusor pressure. It is usually diagnosed by studying the synchronous values of urine flow rate and detrusor pressure and any post‐void residual measurements. A urethral stricture or obstruction due to higher degrees of uterovaginal prolapse or obstructed voiding after stress incontinence procedures is among the possible causes.
- *Dysfunctional voiding*: This is characterised by an intermittent and/or fluctuating flow rate due to involuntary intermittent contractions of the periurethral striated or levator muscles during voiding in neurologically normal women. This type of voiding may also be the result of an acontractile detrusor (abdominal voiding) with electromyography (EMG) or video‐urodynamics required to distinguish between the two entities.

We also looked at the ways that Fowler's syndrome has been defined in the literature, which can be summarised as follows:

- ‘The condition is characterised by a poorly relaxing urethral sphincter that manifests as functional bladder outlet obstruction, associated with an abnormally elevated urethral pressure profile and/or abnormal urethral sphincter electromyography’.^6^
- Retention or obstructed voiding and maximum flow rate (Qmax), which is the highest rate of urine flow achieved during voiding of <15 mL/s with elevated maximal urethral closure pressure and sphincter volume.^7^
- ‘The underlying abnormality was a poorly relaxing rhabdosphincter which when studied using concentric needle electromyography showed a distinct abnormal pattern suggesting direct spread of impulses between muscle fibres’.^8^

We created a combined definition detailed below, which uses the term **chronic idiopathic urinary retention** to refer to idiopathic forms of the condition, and the subset in which a diagnosis of Fowler's syndrome may be reached using electrophysiological and other criteria (Table 1). We recognised that voiding dysfunction exists on a spectrum, and that this definition requires more refinement in the future but considered it necessary for this framework document.

**TABLE 1:** Fife definition of chronic idiopathic urinary retention.

• Urinary retention is defined as a **post‐void residual of >100 mLs,** however, the residual volume seen in chronic idiopathic urinary retention is usually significantly higher.
• Symptoms of urinary retention must be ongoing for at **least 3 months.**
• Retention can be **continuous or intermittent, but recurrent** over a consecutive 3‐month period.
• Requires **catheterisation** to void or has been offered catheterisation to void due to the severity of symptoms.
• Urinary retention is not explained by neurological, urological or gynaecological disorders, which may cause urinary retention.
• Fowler's syndrome is a subset of chronic idiopathic urinary retention, identified on the basis of additional electrophysiological findings.

## 5 AN AETIOLOGICAL AND MECHANISTIC FRAMEWORK FOR UNDERSTANDING CHRONIC IDIOPATHIC URINARY RETENTION

**FIGURE 1:**
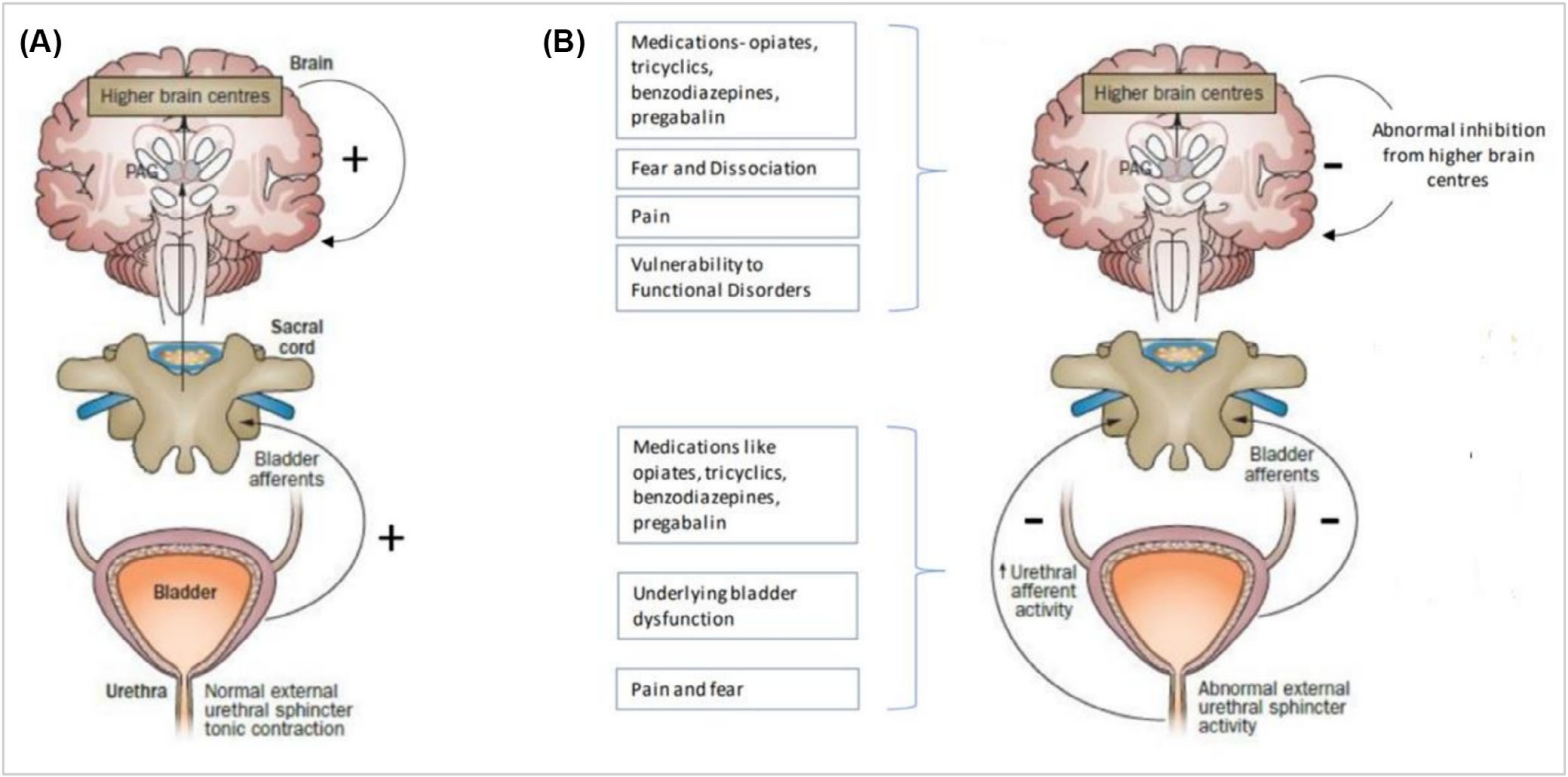
A proposed model of normal and abnormal pathways in brain‐bladder function and processes, which may contribute to urinary retention. Reprinted a/w permission from Springers.

The group considered it important to set out, in brief, a mechanistic and aetiological framework for chronic idiopathic urinary retention to provide a rationale for nonsurgical approaches to treatment (Figure 1).

### 5.1 Normal function

Normal bladder function requires both bottom‐up (afferent signals from the bladder, urethra and pelvic floor) and top‐down (higher level brain function) control of the bladder, with autonomic and somatosensory pathways working in synergy.^9^, ^10^ Peripherally in the lower urinary tract, the internal and external urethral sphincter, bladder and pelvic floor provide information to the sacral area of the spinal cord about bladder fullness. The internal and external urethral sphincter, bladder and pelvic floor ensure continence through parasympathetic and perineal nerve input. As the bladder fills, the bladder muscle relaxes, and the internal and external sphincters and pelvic floor contract as the bladder fills. The pelvic floor and external urethral sphincter are skeletal muscles and are therefore usually under voluntary control (they can be squeezed/relaxed voluntarily in healthy individuals). Relaxation of pelvic floor muscles and the urethral sphincter is the primary phase of micturition and is required to allow detrusor contraction during voiding.^11^ Centrally in the brain, the urination system is mediated by input from frontal brain networks, which assess whether it is safe and appropriate to void, the periaqueductal grey, which sets micturition thresholds (the level at which the person becomes aware of a desire to void) and the pontine micturition centre. The pontine micturition centre is inhibitory, and when safe and socially appropriate to void, it shifts from the storage to the voiding phase.^11^

### 5.2 Dysfunctional voiding and chronic idiopathic urinary retention

Although the pelvic floor and external urethral sphincter are normally under voluntary control, conditions such as dysfunctional voiding illustrate that pathological overactivity in these structures can occur independently of conscious control. Despite the involuntary nature of the urethral sphincter and pelvic floor dysfunction, this overactivity may be modified by peripheral and central treatment (e.g., biofeedback and cognitive behavioural therapy).^12^ It is important to emphasise the lack of willed/voluntary nature of these conditions.

The majority of patients with chronic idiopathic urinary retention have a precipitating event for their retention, commonly surgery, childbirth or a urinary infection.^13^ Current understanding of chronic idiopathic urinary retention is that there is overactivity of the external urethral sphincter and/or pelvic floor. This overactivity also appears to have central effects as it leads to inhibition at the level of the sacral cord, reducing or stopping signals normally sent to alert the pontine micturition centre when the bladder is full. In addition, comorbid pain, dissociation and fear, all forms of fight or flight and ‘threat’ response, are likely to be inhibitory to bladder voiding by impacting on the frontal micturition networks.^14^ Medications for pain, particularly opiates, have both peripheral effects on bladder mechanoreceptors and central inhibitory effects by acting on mu receptors in the spinal cord and periaqueductal grey.^13^

This evidence of peripheral and central interacting systems suggests that chronic idiopathic urinary retention can be viewed using a similar aetiological framework to functional disorders such as fibromyalgia, irritable bowel syndrome and functional neurological disorder. In those conditions there are parallel disruptions in motor and sensory brain networks, especially those involved in bodily agency, attention and emotion.^15^ Functional disorders also involve similar predisposing, precipitating and perpetuating factors to chronic idiopathic urinary retention.

## 6 ASSESSMENT AND DIAGNOSIS

Comprehensive diagnostic‐focused/medical/clinical assessment recommendations have been published elsewhere and inform this multidisciplinary nonsurgical focused paper.^16^, ^17^ There are important differential diagnoses, which include mechanical, neurological and medication/other (see Table 2) and key references ^18^ and^19^. Once other aetiologies of urinary retention have been ruled out, patients fulfilling the Fife definition can be considered to have chronic idiopathic urinary retention.

**TABLE 2:** Conditions to consider and exclude prior to a diagnosis of chronic idiopathic urinary retention.

Category	Specific causes
**Structural (urological/gynaecological)**	
Congenital malformations	Posterior urethral valves
Tumours	Prostate and bladder; gynaecological (e.g., leiomyomas); intestinal (e.g., chronic constipation)
Strictures	Urethral stricture; bladder neck stenosis; urethral diverticulum or cysts; radiotherapy
Calculi	Bladder or urethral calculi
Urogenital prolapse	
**Neurological**	
Spinal cord lesions	Inflammatory, spinal cord injury and degenerative myelopathy tumour
Degenerative conditions	Multiple system atrophy; pure autonomic failure; multiple sclerosis; Parkinson's disease
Sacral Plexus/peripheral nerve pathology	Diabetes, inflammatory/infective (especially HSV‐2) polyradiculopathy, trauma
**Other**	
Pharmacological	Opiates; anticholinergic drugs or those with anticholinergic activity; benzodiazepines

In addition to standard urological, gynaecological and neurological assessment, we recommend considering the assessment within a biopsychosocial framework shown in Table 3. Table 4 sets out how to explore comorbidities including trauma.

**TABLE 3:** Biopsychosocial framework of predisposing, precipitating and perpetuating factors of chronic idiopathic urinary retention.

	Predisposing	Precipitating	Perpetuating
**Biological**	Urinary tract infections Childhood urological issues, e.g., enuresis and urinary tract infections Constipation Medication (especially opiates and benzodiazepines) Caffeine excess or lack of fluid intake Stress incontinence Lack of feeling in the pelvis Pelvic pain conditions (endometriosis, irritable bowel syndrome, interstitial cystitis, vulvodynia, pudendal neuralgia, etc.) Connective tissue disorders (e.g., hypermobility/Ehlers‐Danlos syndrome type 3) Difficulties getting on and off the toilet independently	Urinary tract infection Surgery due to either to the severe local pain or anaesthesia causing dissociation Childbirth (with or without pelvic trauma) Any pelvic inflammatory process/condition Lumbopelvic trauma Pelvic pain Illicit substances	Recurrent urinary tract infections Ongoing pelvic floor high tone Constipation and IBS Medication (especially opiates and benzodiazepines) Persistent poorly controlled pain Caffeine excess Lack of fluid intake Difficulties getting on and off the toilet independently
**Psychological**	Psychiatric symptoms (symptoms of anxiety, low mood, irritability and mood swings, compulsive behaviours and intrusive thoughts) Psychiatric disorders (Severe and diagnosable generalised anxiety or panic disorder, depression, obsessive compulsive disorder and complex‐PTSD) Chronic/nociplastic pain (may be considered as both a biological and psychological factor) Trauma (interpersonal and/or sexual) Fear of toileting or feeling dirty Avoiding toileting when out of the home—‘holding on’	Feeling of being out of control Panic or dissociation An aversive conditioning event, e.g., an unexpected episode of bladder dysfunction and associated feelings of shock/embarrassment	Not being believed/being dismissed by caregivers including clinicians Difficulty navigating work, life, relationships, sex with retention or need to catheterise Panic and dissociation Alexithymia or emotional dysregulation Attentional bias to symptoms/affected area, symptom‐monitoring behaviours and cognitions (including fear of toileting or feeling dirty) Beliefs and expectations about illness Avoidance‐based coping External locus of control Unhelpful safety behaviours e.g. avoiding fluids to avoid the need to void
**Social**	Infrequent voiders, e.g., shift workers, schoolchildren, those with lack of access to clean toilets or privacy Lack of information about normal toileting and good basic bladder advice Avoiding toileting when out of the home—‘holding on’	An event when access to an acceptable toilet was prevented, e.g., shift workers, schoolchildren, those with lack of access to clean toilets or privacy Lack of support pre and post childbirth	Infrequent voiding behaviour/habits Lack of easy access to acceptable toilets Difficulties getting on and off the toilet independently Feeling of lack of or incomplete privacy? (eg toilets in open space/small space where worried people can hear)

**TABLE 4:** Common comorbidities influencing bladder function and suggested open‐ended questions.

Domain influencing bladder function	Relevance to bladder dysfunction and examples of open‐ended questions to explore this area
Daily activity, lifestyle and participation	Screen for biopsychosocial factors that may affect bladder dysfunction, including mobility, difficulties with activities of daily living, social history, etc (see Table 2) Please describe a typical day. Is there anyone at home with you? Do you need any help from others during the day? Do you worry about leaving the house and the need to use the toilet when out? Are you able to prepare for yourself a meal and a drink/hot drink?
Nutrition and hydration patterns	Screen for atypical eating habits, avoidance or insufficient fluid intake, or reliance on caffeine or energy drinks that may irritate the bladder. Tell me about your usual eating patterns over a typical day. ‘Are their times when it feels harder to eat or drink, or reasons you might limit your fluid intake?’ How much do you drink in day? What do you drink? Do you drink caffeinated drinks?
Toileting habits and beliefs (including public toilets)	Explore frequency of toileting, avoidance of public toilets/holding on, social or hygiene‐related fears and phobias of public toileting. ‘Can you describe your current toileting routine? And how has this changed over time?’ ‘What is your experience of using toilets away from home?’ or ‘Are there any worries or beliefs about using the toilet that affect what you do?’
Pain	Explore the impact of pain on usual activities and toileting. Is the onset of pain related to the bladder, other pelvic symptoms or specific trauma? Do you experience regular pain, tell me about the pain you experience. What impact does pain have on your bladder symptoms and toileting
Mood, anxiety and sleep	Explore the relationship between mental and physical health Have your symptoms/bladder dysfunction affected your mood? How have your mood and energy levels been recently? Tell me about your sleep? Do you have difficulty getting or staying asleep? How is your memory and concentration? How does anxiety or stress tend to show in your body?
Trauma and adverse life experiences	Give the patient an opportunity to disclose recent or past trauma. Sometimes trauma or abuse of any kind can be relevant to bladder dysfunction, is this something that is relevant to you? Some people find that difficulties or overwhelming experiences earlier in life can affect their health later. Is there anything from your past which you think might be relevant to your health now? Has anyone ever touched you in a way that you did not wish to be touched? Is there anything you would like me to be aware of that might influence how your body responds to stress or symptoms? Be prepared to follow up with an appropriate response, e.g. Thank you for trusting me with this information. If there is an ongoing safety issue, follow the appropriate local policy. Consider having local signposting/information available in clinic to provide to patients. This might be relevant to your current health problems. Have you ever spoken to anyone about this? If not, how would you feel about talking to someone? This allows the clinician to make a referral to a healthcare professional/psychologist/psychiatrist if the patient is receptive and would like to explore this or to revisit later if the patient does not feel ready or able to explore this.
Functional symptoms/disorders and persistent physical symptoms	People with chronic idiopathic urinary retention commonly have other functional symptoms, and specialist diagnosis and treatment may have a positive impact on bladder function. Have you ever been given explanations for symptoms that did not show up on tests? Have you ever seen a neurologist because of unexplained weakness, fits or seizures, persistent fatigue, speech or swallowing problems? Have you ever seen specialists for symptoms such as bowel changes, pain, breathing, weakness or seizures?
Joint hypermobility and connective tissue issues	Joint hypermobility spectrum syndromes commonly coexist with functional symptoms. Do you have very flexible joints now, or when you were younger? Do you experience frequent joint injuries or dislocations?
Neurodiversity and sensory processing	Neurodiversity, autism spectrum disorder often coexists with functional symptoms (and hypermobility). Recognition of coexisting problems may help to provide more personalised treatment approaches. Have you ever been diagnosed with ADHD or autism? Or explored if neurodivergence might be part of how your brain works? Do you have any sensory sensitivities that influence toileting, bladder awareness or routines? Did you meet your developmental milestones as a child?
Beliefs, understanding and expectations about symptoms	Explore beliefs, concerns and expectations in order to provide personalised education and information about the diagnosis and address specific fears or beliefs that may be barriers to engagement with treatment. What do you think might be causing your bladder problems? What explanations have others suggested to you? Including family and friends. Are there any unanswered questions or concerns about your diagnosis? What kinds of tests or treatments do you feel might still be missing? Are there any treatment that you think might help you?

## 7 TREATMENT

We recommend a stepped treatment approach starting with early or immediate access to basic interventions that can be implemented by nonspecialist clinicians. The complexity of care involving clinicians with specific training and experience in bladder dysfunction increases according to patient need in subsequent steps. We have summarised these treatments in Figure 1. The proposed stepped model includes the following stages:

- Step 1: Explanation and formulation
- Step 2: Basic bladder healthcare
- Step 3: Optimising catheterisation
- Step 4: Management of relevant comorbidities
- Step 5: Multidisciplinary treatment
- Step 6: Additional treatment options and emerging therapies

### 7.1 Step 1: Explanation and formulation

**FIGURE 2:**
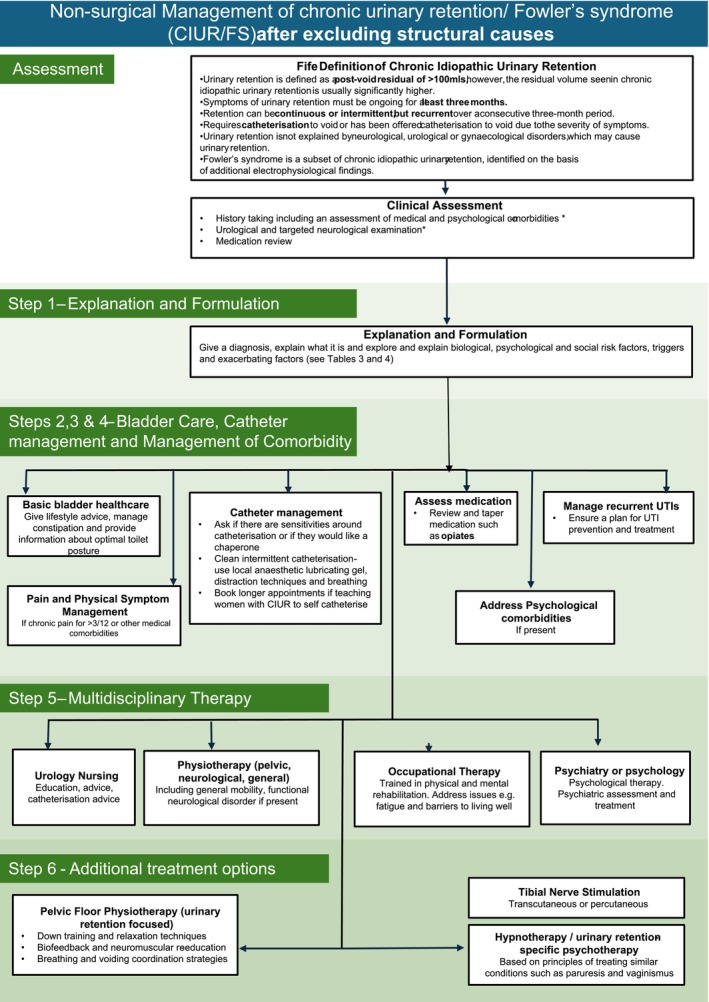
Framework algorithm of nonsurgical treatment.

Education about the nature of the disorder is an essential platform for further treatment. Clinical experience suggests that the more the patient understands, the more likely they are to engage with treatment. Many patients gain the impression that their condition is completely unrecognised and mysterious, when that is not the case. A tailored explanation may involve some of these steps:

1. Give a diagnosis—for example, chronic idiopathic urinary retention or Fowler's syndrome.
2. Explain that this is a well‐recognised situation familiar to the treating team.
3. Explaining that it is a bladder‐brain disorder in which the main problem is abnormal bodily functioning rather than damage, so there is potential for improvement.
4. Consider the use of metaphors and comparisons, for example:
  - ‘Some people have difficulty peeing when they have a bad urinary tract infection or severe back pain because it's so uncomfortable. Chronic idiopathic urinary retention is like this, but the muscles get stuck in spasm. Having severe pain (even once), recurrent infections or feeling unsafe can have a longer term effect on the ability to urinate’.
  - ‘Your brain, bladder and pelvic floor have to work together for you to be able to urinate. Just now those connections aren't working well’.
  - ‘There's a problem with the automatic control between brain, pelvic floor and bladder. It's like a software problem in your body, rather than broken hardware. This means that perhaps we can help retrain your bladder with specific techniques’.
  - ‘At the minute when you are trying to pass urine, it's like a dishwashing liquid bottle or water bottle being squeezed with the cap on. The pressure is building up, and the liquid has nowhere to go. Your bladder is trying to push against a urethral sphincter and pelvic floor that aren't relaxing’.
5. Explain that a wide variety of factors may be involved (see formulation below)
6. Explain how various types of intervention (see Figure 2) may improve symptoms by improving functioning. More detail in Step 2.
7. Provide written information where possible, including copying correspondence to the patient as well as online resources for information and patient‐led organisations (e.g., www.fowlerssyndrome.co.uk or https://neurosymptoms.org/en/symptoms/fnd-symptoms/bladder-symptoms-and-fnd/) (for those individuals with comorbid FND).
8. Start the process of ‘formulation’. A formulation is a way of bringing together all the relevant factors for that individual to help the clinician and patient understand ‘why’ and ‘how’ symptoms have happened (Table 3, supplementary cases). A formulation would often be done by a psychologist, psychiatrist or allied health professional collaboratively with the patient, but it can help if the clinician making a diagnosis can begin that process. Surgeons and physicians may want to focus especially on how urinary retention has occurred in the body but leaving space for ‘why’ questions to be addressed at a later date. Formulation can help build confidence in the diagnosis, bringing together strands of someone's own history in a way that provides personally meaningful context to their symptoms. It can also offer potential for improvement by showing how treating coexisting problems, self‐management strategies, physiotherapy and other treatments can help.

### 7.2 Step 2—Basic bladder healthcare

Many simple issues will exacerbate chronic idiopathic urinary retention. Getting the basics right may not resolve the issue of chronic idiopathic urinary retention but will provide a good baseline to start treatment. Additionally, basic changes (e.g., drinking adequately) may be necessary for the patient to benefit from more specialist treatment. Supporting patients to get the basics right and optimise bladder health does not require specialist training or qualification. We therefore suggest that all qualified members of the multidisciplinary team (nurses, allied health professionals, surgeons and physicians) can participate in promoting basic bladder health.

#### 7.2.1 Lifestyle issues—Hydration, caffeine, exercise, sleep

We recommend that patients drink 1.5 to 2 L of fluid a day with the majority of fluid intake coming from water. Many patients lower their fluid intake to reduce incontinence or to avoid having to catheterise outside the house. However, poor intake results in increased urine concentration and this is irritating to the bladder. Alcohol and caffeine are also bladder irritants and should be minimised or avoided where possible. Optimising sleep and having small amounts of gentle regular exercise (three or more times per week) are likely to be helpful for good bladder function.

#### 7.2.2 Managing constipation

**FIGURE 3:**
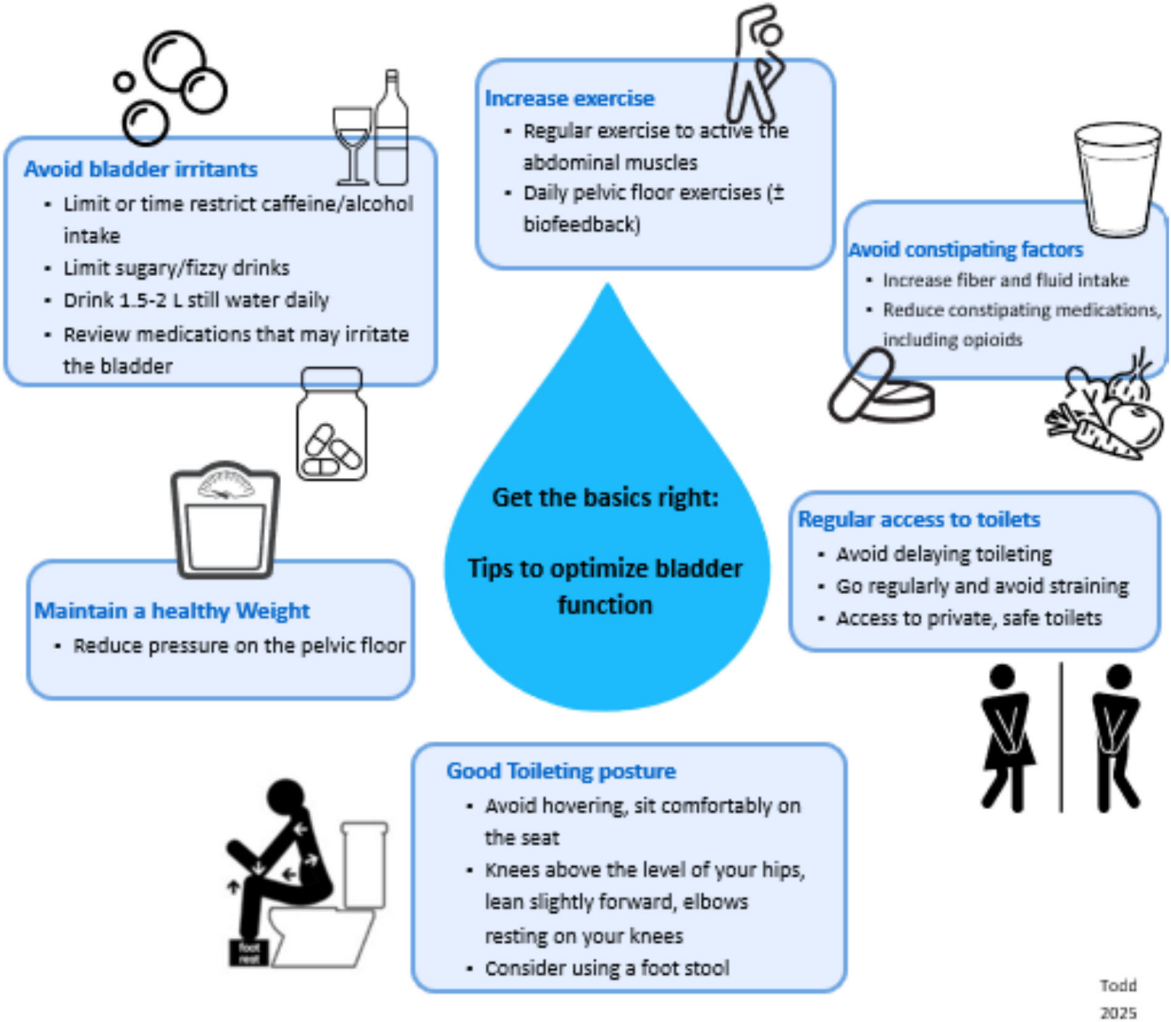
Basic bladder health is often missed for women with complete urinary retention.

Patients should aim for regular bowel motions. Ask the patient about their normal bowel pattern and aim to optimise bowel consistency to Bristol stool chart Types 3, 4 or 5 through diet and/or medication. Avoid constipation, discuss bowel mechanics to avoid straining and discuss appropriate positioning (see Figure 3). Many patients will skip meals or eat little, particularly if they have coexisting severe pain. Encourage regular food intake, even if this starts out with small amounts. A high fibre diet which has fruit, vegetables and seeds will help with constipation. A healthy, varied diet containing both soluble and insoluble fibre will help with constipation. Simple changes like switching from white bread and pasta or rice to wholemeal or brown options and adding more fruit, vegetables, pulses and seeds can be helpful. However, if fibre needs to be increased, this should be done over the course of a few weeks, not overnight. And adequate fluids are essential—increasing fibre without adequate fluids can make constipation worse.

#### 7.2.3 Toileting posture

Where patients can pass urine intermittently, optimising toileting posture is encouraged to facilitate gentle pelvic floor relaxation. Patients should have their knees slightly higher than their hips; placing their feet on a small footstool may be helpful with this.

### 7.3 Step 3: Catheter management: How to get it right first and every time

Catheterisation is often more traumatic for women than many in urological and general medical services recognise. Consider whether there may be sensitivities around examination or catheterisation or previous traumatic events that require a female member of staff or a chaperone. A chaperone can provide distraction techniques and provide comfort and a feeling of safety. Catheterise in a sensitive way, aiming to reduce trauma. For example, we strongly discourage staff using their mobile phone light to locate the urethra. For good technique, use analgesia and numbing agents and ensure adequate time for the numbing to work (at least 5 min). Be aware that there might be urethral or pelvic floor spasm, which should be reduced by analgesia and numbing. Ask patients to take deep, out breaths (‘breathe in and now breathe out for 1,2,3, 4’), while the catheter is being inserted. Alternatively, try distraction techniques such as talking about their best holiday, putting on the TV, radio or a podcast or asking them to hum a tune or make a deep ‘moo’ noise. This helps to relax the pelvic floor.

Consider booking a longer appointment when teaching women with chronic idiopathic urinary retention how to intermittently self‐catheterise, as spasm may make the experience more frightening and unpleasant. Having enough time to work slowly through the education and techniques will allow a more positive experience for both patient and staff.

Patients who need to catheterise recurrently should be assisted in getting access to disabled toilets, for example, in the United Kingdom, using the RADAR key to enable them to have access to private disabled toilets. Patients should also be offered a range of intermittent self‐catheters to ensure the best fit for the individual patients' needs and lifestyle. For patients with an indwelling catheter, consider a catheter valve as opposed to free drainage into a leg bag.

### 7.4 Step 4—Optimising relevant comorbidities

We suggest a wider focus than just the bladder. Helping patients with chronic idiopathic urinary retention to understand how their various comorbidities may link to their condition can be a ‘lightbulb’ moment for some individuals and their families. There may be therapeutic opportunities to reduce harmful medication or address comorbidities, which may be perpetuating their bladder symptoms.

#### 7.4.1 Tapering harmful medication

Medical oversight is required for tapering medications and diagnosing and treating comorbidities. All patients should have pain medications optimised. Opiates, benzodiazepines and tricyclics will have an adverse effect on bladder as well as bowel function and are often not indicated for long‐term treatment. Most patients with chronic idiopathic urinary retention will have primary or nociplastic pain.^20^ Recent guidelines, such as NICE guidelines,^21^ have suggested that gabapentinoid and opiate medications are unhelpful in chronic primary pain. Ask about the duration of medications for pain such as opioids, benzodiazepines or tricyclics; if longer than 3 months, do they feel that the medications are helping with pain, function, mood, sleep and quality of life? Is the patient's pain severity still rated as high? Are there any side effects—concentration, mood, somnolence, constipation and poor memory? If so, this could lead to a conversation about gradual tapering with minimal side effects which frequently leads to an improved quality of life.^22^ Medication tapering works best when the patient themselves has decided that they wish to do it and has had input into the speed of tapering, since pain and other symptoms may flare during and after the process.

#### 7.4.2 Optimise treatment in recurrent urinary tract infections

Urinary tract infections are a common triggering event for patients with chronic idiopathic urinary retention and recurrent urinary tract infections can be a perpetuating factor for some patients. If there are recurrent urinary tract infections (European Association of Urology definition two or more episodes of symptomatic UTI within 6 months, or 3 or more UTIs within 12 months^23^) refer for urology input. Assist patients with understanding the difference between colonisation and infection.

#### 7.4.3 Address comorbid chronic pain and other physical symptoms

Chronic pain is present in up to 75% of patients^3^ with chronic idiopathic urinary retention and nonpharmacological treatment of chronic pain is often a major unmet need. Review any medications prescribed for pain (as above) and suggest or make a referral to pain management services for holistic care if possible. Rehabilitation therapy is now regarded as the gold standard treatment for chronic pain, not medication.^21^

Recognising links to hypermobility spectrum disorders can be helpful to aid understanding, as it is a common link between symptoms and syndromes that often cluster together, such as anxiety, autism spectrum disorder, functional neurological disorder, sensory processing difficulties, irritable bowel syndrome, bladder and other somatic symptoms. Emphasising that this is not an obstacle to improvement is an important step, and there may be a role for physiotherapists and occupational therapists to optimise joint stability and protection. Making links or integrating patients' understanding of their conditions can help make sense of what may seem a bewildering list of conditions.^24^

#### 7.4.4 Address comorbid psychological disorders

Psychological disorders such as PTSD, anxiety disorders and depression affect a large proportion of patients with chronic idiopathic urinary retention. Based on our understanding of the effect of central mechanisms on the ability to void, and the shame and stigma associated with bladder dysfunction, it is not surprising that patient's mental, as well as physical health will impact on their bladder function, or that mental health difficulties can arise as a consequence of living with a debilitating condition. If symptoms are affecting the patient's ability to engage fully with life, consider onward referral to specialist mental health services (psychiatry, clinical psychology, psychotherapy, pain management service, chronic fatigue services and adult mental health services).

### 7.5 Step 5—Multidisciplinary treatment

#### 7.5.1 Urology/continence nursing

Urology nurses are key to providing catheter care education, promoting independence with catheter management, including comfort and positioning for self‐catheterisation and posture and ensuring that patients have access to supplies. They can explore patient thoughts and feelings about catheter use. Some patients develop a phobic anxiety around catheterisation due to pain or unpleasant experiences of catheterisation, especially if there is a history of previous trauma. Additionally, urology nurses will have experience of supporting patients with recurrent urinary tract infections and knowing when urological re‐referral or assessment is required. They will often be the first port of call for patients who are struggling with their urinary retention. Urology nurses can also offer patients advice on managing intermittent self‐catheterisation in situations, which may be uncomfortable for patients to bring up by themselves, for example, around menstruation.

#### 7.5.2 Physiotherapy (neurological or MSK)

Standard approaches to pelvic physiotherapy can help with a range of issues experienced by patients with chronic idiopathic urinary retention, such as overactive bladder or pelvic pain. Physiotherapy and occupational therapy may be required to optimise physical access to toileting in patients with chronic idiopathic urinary retention and any of the common comorbidities, such as functional neurological disorder, joint hypermobility or chronic pain. This can be achieved by improving transfers and mobility, as well as optimising toileting posture. Certain body positions and stretches performed before voiding can help release tension in the pelvic floor and promote relaxation, including yoga poses such as *happy baby* and *child pose*. Physiotherapists may also teach diaphragmatic breathing techniques, which help synchronise movement of the diaphragm with the pelvic floor and activate the parasympathetic nervous system, promoting relaxation of both the pelvic floor and the body as a whole.

In patients with functional neurological disorder, bladder function may improve in tandem with successful rehabilitation of functional motor symptoms. Movement retraining with diverted attention can be delivered according to consensus recommendations in FND physiotherapy.^25^ For individuals with generalised pain, physiotherapy treatments that lead to overall pain reduction can anecdotally help with pelvic tension and bladder function.

#### 7.5.3 Specialist pelvic physiotherapy directed at chronic urinary retention

Pelvic health physiotherapists can use a range of strategies to support people with chronic idiopathic urinary retention. Although no retention‐specific physiotherapy protocol currently exists, management draws on approaches used for neurologically intact patients with voiding dysfunction, pelvic floor or urethral sphincter overactivity, pelvic pain and associated bladder, bowel and sexual symptoms. These interventions are commonly used in secondary care following specialist assessment.

Treatment should be delivered by a specialist pelvic health physiotherapist and guided by a detailed assessment of pelvic floor function and relevant extra‐pelvic factors, including breathing pattern, abdominal wall function, lumbar spine, pelvis, hips, fascia and wider musculoskeletal contributors.

Physiotherapy usually begins with education and explanation, often supported by a pelvic model to demonstrate pelvic floor anatomy. Relaxation‐based pelvic floor retraining may be most helpful in patients with global pelvic floor overactivity, rather than isolated urethral sphincter overactivity. The aim is to optimise pelvic floor function by improving relaxation, range of motion, coordination and, where appropriate, strength.

Behavioural and environmental factors should be optimised, including toilet posture and toileting habits. Stretches or positions before voiding, such as child's pose or happy baby, may help reduce pelvic floor tension. Diaphragmatic breathing can also be taught to coordinate diaphragm and pelvic floor movement, promote parasympathetic activity and support whole‐body relaxation. Double voiding may be helpful for some patients with pelvic floor overactivity.

Chronic idiopathic urinary retention may also be associated with reduced body awareness, particularly in the context of trauma, shame related to urination, pain or prior medical procedures. In these cases, early treatment may focus on improving body awareness and tolerance of bodily sensations before progressing to specific pelvic floor retraining.

Pelvic floor exercises can be adapted for retention by emphasising relaxation and release rather than strengthening. Digital tools, such as pelvic floor apps, may be used in this way where appropriate. EMG biofeedback has evidence in dysfunctional voiding and may be helpful when patients have difficulty identifying pelvic floor contraction and release.

Selected patients with myofascial pain, hypertonia or poor pelvic floor release may benefit from intravaginal or intrarectal manual therapy. This can include superficial and deep pelvic floor myofascial release, combined with education, graded exposure to sensation and movement, stretching, joint mobility exercises and strategies to reduce threat and restore normal pelvic floor function. Patients may also be taught safe self‐release techniques using a wand or dilator at home.

Treatment should be individualised and developed collaboratively with the patient.

#### 7.5.4 Occupational therapy

As occupational therapists are trained in both physical and mental health rehabilitation, they are well placed to take a holistic approach to care. This enables exploration of how chronic idiopathic urinary retention affects not only physical functioning but also relationships, everyday roles, self‐identity and body image. Occupational therapists can identify barriers to participation in daily activities that arise from toileting difficulties and work collaboratively with individuals to develop practical strategies to reduce these barriers. They can also recognise when reduced activity or loss of meaningful roles is contributing to increased symptom focus and support re‐engagement in valued occupations to promote wellbeing and participation. Occupational therapists can promote and facilitate regular movement, such as sitting out of bed, changing posture regularly, mobilising when able and engaging in standing activities, to support physical wellbeing and minimise activity avoidance. When pain and fatigue significantly affect daily functioning, they can support people to pace daily activities and routines, helping them manage energy levels, maintain good bladder care habits and avoid unhelpful ‘boom and bust’ patterns of activity.

#### 7.5.5 Psychological therapy

Psychological difficulties might arise because of having the disorder and/or might be contributory factors to symptoms of chronic idiopathic urinary retention. While the frequency of psychological/psychiatric morbidity is not widely studied in patients with chronic idiopathic urinary retention, a recent study of patients with Fowler's syndrome found high rates of psychological distress and trauma (97%), with 71% having a psychiatric comorbidity and 59% having a functional neurological disorder, which may also benefit from psychological therapy.^26^

Psychologists and psychiatrists can support patients with chronic idiopathic urinary retention by assessing and formulating potentially relevant individual psychological factors, such as fear and avoidance, and providing treatment for psychological comorbidities when indicated. Clinically, we see patients who develop a phobia to intermittent self‐catheterisation, but this is not well recognised in the literature. Patients with this phobia may, in our experience, benefit from a CBT approach.

We propose that patients with chronic idiopathic urinary retention may also benefit from access to established psychological therapies that are routinely used to treat related or co‐occurring conditions such as depression, anxiety disorders and post‐traumatic stress disorder. These evidence‐based therapies include cognitive behavioural therapy, acceptance and commitment therapy, trauma‐focused interventions and compassion‐focused therapy. Many of these psychological therapies are also helpful in treating comorbid pain conditions, or other functional disorders.

Some patients may benefit from specialised psychiatric input, especially in the presence of more complex or severe difficulties that require pharmacological therapies. Such medications can used alone or often as a helpful adjunct to psychological therapy to treat existing morbidity, rather than the symptom of retention itself.

### 7.6 Step 6—Additional treatment options and promising therapies

The consensus group considered a range of more advanced, condition‐specific therapies for urinary retention, for which the current evidence base is limited.

#### 7.6.1 Transcutaneous tibial nerve stimulation (TTNS)

TTNS used at the medial malleolus is already used in patients with overactive bladder syndromes and there are case reports of its use in patients with idiopathic urinary retention.^27^, ^28^, ^29^, ^30^ As a noninvasive and inexpensive treatment, it could also be considered as a treatment adjunct.

#### 7.6.2 Hypnotherapy/urinary retention‐specific types of psychotherapy

Hypnotherapy has an evidence base in the management of functional gastrointestinal disorders^31^, ^32^and a more limited one in urgency urinary incontinence/overactive bladder.^33^ Chronic idiopathic urinary retention is a problem that on theoretical grounds could be a good target for a specific hypnotherapeutic approach.

#### 7.6.3 Psychological therapy tailored to urinary retention

Earlier literature includes case reports and small case series describing women with chronic urinary retention who improved following psychological therapies delivered by psychiatrists or psychologists. While the evidence base is limited, it seems reasonable to draw cautious inferences from evidence relating to the treatment of closely related conditions. Studies of cognitive behavioural therapy often incorporating behavioural therapy and combining graded exposure techniques have moderate to strong evidence in vaginismus, a condition where there is similar excessive muscle contraction in the pelvis.^34^ Similarly, psychotherapy is effective for paruresis^31^, ^32^, ^35^ and also appears to be effective in overactive bladder in combination with bladder retraining.^36^

Psychotherapy for chronic idiopathic urinary retention could incorporate elements of treatment from these other conditions. Clinical experience suggests that a trauma‐focused component will be important for a subset of patients. The key difference to psychological therapy described in Step 3 is that the therapy would be designed to improve the symptom of urinary retention itself and not just manage psychological comorbidity.

## 8 DISCUSSION

There is little information on how to treat chronic idiopathic urinary retention. We have created an algorithm to demonstrate how this multidisciplinary framework could be aligned to normal clinical practice (Figure 2, clinical cases in Supporting Information).

Many women with chronic idiopathic urinary retention are currently told that they have an ‘atonic bladder’ or that their problem can only be managed invasively. This framework aims to identify practical strategies to help manage symptoms and enhance quality of life and care for patients with chronic idiopathic urinary retention. It also provides potential ideas for more specialist women's health physiotherapy and psychological therapy, ideally in combination, which may have a role to play. This framework also highlight the large gaps in our knowledge for these severely affected patients.

Limitations include the limited primary evidence base for many interventions used in chronic idiopathic urinary retention. Detailed discussion of pharmacological treatments, including alpha‐blockers and other medications with limited evidence, was beyond the scope of this paper, which focused on multidisciplinary assessment and first‐line nonsurgical management. We also did not aim to define a consensus urodynamic classification of chronic idiopathic urinary retention but instead focused on practical clinical management after specialist evaluation. Comments on quality of life are informed by clinical experience and the broader literature rather than validated condition‐specific assessment tools.

Future research including a broader comparison of the literature assessing pros and cons of all treatments for chronic idiopathic urinary retention and consensus on how and when rehabilitative strategies should be integrated into clinical care pathways is required. These issues and creation of research questions will be addressed in forthcoming work done with Fowler's Syndrome UK and international urological, neurological, obstetrics and AHP colleagues in the Consensus Guidelines on chronic idiopathic urinary retention/Fowler's syndrome: assessment, treatment and education (CURATE) project.

## AUTHOR CONTRIBUTIONS

All authors contributed meaningfully to discussion and/or paper write up.

## DISCLOSURE

IH, LMcW, AC and JS all provide expert opinion in medicolegal cases. IH and JS have research funding from the Chief Scientist Office of Scotland. JP and GN have research funding from the NIHR. IH, JP, HS and JS are trustees on the Fowler's Syndrome UK board. All other authors report no disclosures.

## CONFLICT OF INTEREST STATEMENT

JS, IH, AC and LMcW do expert witness work. IH and JS are supported by NRS Fellowships by Scottish government funding. JP is supported by NIHR funding and does private work. JS, IH and JP have had unrestricted funding for an educational meeting from Medtronic and The Urology Foundation which was used to run the Consensus Guidance on Chronic Idiopathic Urinary Retention/Fowler's Syndrome: Assessment, Treatment and Education (CURATE) conference in February 2026.

## Supporting information


**Data S1.** Supporting Information.
