# Supplementary material for: Chronic idiopathic urinary retention and Fowler's syndrome in women: A multidisciplinary framework for assessment and nonsurgical management
Source: BJUI Compass. 2026 Jul 2;7(7):e70240. doi: 10.1002/bco2.70240 (PMC13325401; doi:10.1002/bco2.70240)
Supplement: Supplementary file 1 — Data S1. Supporting Information. [file BCO2-7-e70240-s001.docx]

**Supplementary Information**

**Case studies**

Case studies will be used to highlight typical presentations of patients with chronic idiopathic urinary retention.

| Case study 1 | Potential opportunities to improve care |
| --- | --- |
| A 21 year old lady, Susan, attended the emergency department because she had been unable to pass urine for more than 16 hours. She had a history of back pain which had been increasing over the last few weeks and on the advice of her doctors she had increased her medications to ensure she could still manage to get to work.  On the day in question her back pain was very bad, she’d been unable to pass a bowel motion and when she arrived in the emergency department, staff inserted a catheter and it drained a litre. Both Susan and the emergency department staff were worried by this as she had felt full with some abdominal pain but didn’t realise her bladder wasn’t working to this extent. The catheter insertion was painful, and it felt like the nurse who inserted it was pushing against something. People kept telling Susan to relax during the procedure. Quite apart from the pain of the catheter the insertion happened in the busy emergency department and Susan was worried the curtain would open and everyone would see into her room. The emergency department team were worried she might have a compression on her nerves or spine which would need an urgent operation. She was rushed through for an examination, including a rectal exam, and then had an MRI scan. The MRI scan was normal. No one was sure what this could be when the scan was normal. Susan had the catheter left in and was told to attend for an appointment in six weeks to see if the catheter could be removed. In that time, Susan, who had recently met a new partner, felt very self-conscious. Her catheter leaked urine, and she had to get antibiotics from the GP for an infection. When Susan eventually saw the nurse specialists, they told her they couldn’t assess her but removed the catheter and offered to teach her intermittent self-catheterisation. When she couldn’t pass urine again 24 hours later Susan again had to attend the emergency department. She was referred to urology but after doing an internal examination she was told that there was no reason for her bladder issues.  The staff also said that she shouldn’t be taking the opiates she’d been prescribed because they would be making her constipation worse. No one had told Susan that medications could affect her bladder or bowels before, and she felt ashamed and upset as it felt like the team were suggesting she was to blame. The second time the catheter went in was again really painful and Susan was told that she’d have to learn to self-catheterise while she waited for the investigations.  She felt hopeless and worried. The urodynamic tests were eventually carried out.  These showed overactivity of the urethra but no structural reason for the blockage. She was told her issue was ‘idiopathic’ and no one knew what was wrong. One of the doctors told her she was wasting their time when she attended again out of hours and people couldn’t understand why she couldn’t just learn to self-catheterise.  **Early discussion of likely diagnosis** if no structural cause found. Early referral for pain management and exploration of other comorbidities. Consider MDT referral- physiotherapy, OT, psychology, psychiatry.  **Optimise self- catheterisation**: position, fear, pain and supplies and provide access to disabled toilet key to enable patients to access clean and large toilets.  **Refer to specialist pelvic health physiotherapists**.  **Telling people you believe them** is important for patients who feel disbelieved and stigmatised. Remember the patient is there because they are suffering. | **Constipation:** Will make bladder function worse.  **Early history to identify predisposing and precipitating factors:** (medications, pain, urinary tract infections) and early removal of catheter if no clear aetiology for retention.  **Refer to urology if >1 episode of urinary retention** to enable faster diagnosis to limit number of urinary tract infections and optimise quality of life.  **Optimise first catheterisation experience:** A catheter was needed but using a lot of instillagel and giving it time to work, trying breathing exercises or distraction techniques and trying to ensure the patient feels safe will all make the initial catheterisation less unpleasant .experience.  **Education:** Whilst an MRI is needed in this situation in >70% of cases it will be normal or non-explanatory. Most of the time pain, medications, constipation and bladder underactivity will be causing the symptoms. Patients need to know this before the scan.  **Medications:** Be aware of the impact of medication increases on bladder function. Consider non opiate medications (including avoiding tramadol, codeine and co-codamol). |

| Case study 2 |
| --- |
| Mary had always been a bit hesitant about using toilets that weren’t her own. She liked to be sure that they were clean and private. This had been an issue for her at school when she sometimes had to hold on all day. However, she’d been working for several years, and her bladder issues hadn’t been a problem for a long time. Mary became pregnant and was excited to be a mother. Her pregnancy went well. The labour was difficult and incredibly painful and then the baby got stuck. She was rushed for an epidural and then a forceps delivery. That bit of labour was a bit of a blur, it seemed like things were hard but ok and then suddenly there were so many people in the room, and she was being asked questions, had to sign a form and felt a bit far away and people were saying the baby’s heart rate wasn’t right. She had a significant tear and after the epidural wore off her vulva and perianal region were really sore, although her legs felt back to normal. She found it really difficult to even sit on a chair or the toilet and the idea of passing urine or moving her bowels was quite scary. Despite the pain, Mary was delighted to meet her child. Later that evening the midwives came to check on Mary and her daughter and asked Mary how many times she’d passed urine. Mary realised she hadn’t passed urine since she had the epidural even though she’d gone to try twice. The midwives looked worried and got an ultrasound scanner. Mary had almost a litre of urine in her bladder. Everyone was quite worried about what might have caused this. The doctor came, checked her over and said she needed a scan and a catheter. The scan came back normal, but when they tried to remove the catheter Mary couldn’t pass urine. No one knew what was wrong. It was overwhelming for Mary, she was managing a new baby, a difficult birth with pain and now her bladder didn’t work. Urology came but said it should get better by itself. It didn’t and after two months of ongoing retention no one knew what to do with her. She started getting recurrent urinary tract infections. The cycle of antibiotics, pain, and not knowing made her life smaller and more difficult. Mary wished she could be well and be the mum she wanted to be. She had a catheter in all the time because when she tried to intermittently self-catheterise it was sore and almost impossible to find toilets that were clean where she could put the equipment she needed to catheterise. A few times people had said she shouldn’t be using the disabled toilets because there was nothing wrong with her. Often the disabled toilets were locked. Mary gave up trying to intermittently self-catheterise. When she went out, she did not drink anything in case her catheter leaked. It took three years for Mary to get a name for her condition. The fourth urologist she saw had met others like her and suggested she get an operation. This worked a bit, and she could get rid of the catheter, but Mary still had regular infections, did not leave the house much and when she did, never ate or drank anything out of embarrassment about needing to use the disabled toilets.  **Optimisation of early analgesia** especially after anaesthetic to enable bowel and bladder positioning and emptying.  **Early discussion of likely diagnosis** if no structural cause found. Early referral for pain management and exploration of other comorbidity. Consider MDT referral- physiotherapy, OT, psychology, psychiatry.  **Optimise self catheterisation position:** fear, pain and supplies and provide disabled toilet key to enable patients to access clean and large toilets.  **Basic information:** encourage regular intake to avoid bladder hypersensitivity.  **Early discussion of likely diagnosis** if no structural cause found. Early referral for pain management and exploration of other comorbidity. Consider MDT referral- physiotherapy, OT, psychology, psychiatry.  **Refer to specialist pelvic health physiotherapists**. |

**Optimise self catherisation position:** fear, pain and supplies and provide access to disabled toilet key to enable patients to access clean and large toilets.

**Refer to specialist pelvic health physiotherapists**.

**Early history to identify predisposing and precipitating factors** (medications, pain, urinary tract infections) and early removal of catheter if no clear aetiology for retention.

**Education:** Whilst an MRI is needed in this situation in most cases it will be normal or non-explanatory. Most of the time pain, medications, constipation and bladder underactivity will be causing the symptoms. Patients need to know this before the scan.

**Bladder information:** encourage regular intake to avoid bladder hypersensitivity.

**Early discussion of likely diagnosis** if no structural cause found. Early referral for pain management and exploration of other comorbidity. Consider MDT referral- physio, OT, psychology, psychiatry.

**Optimise self catheterisation position:** fear, pain and supplies and provide Radar key to enable patients to access clean and large toilets.

| Case study 3 |
| --- |
| Simone had severe weakness from a functional neurological disorder. She was admitted to hospital when she had a terrible headache with a spreading odd feeling of tingling coming down the right side of her body. The tingling feeling lasted 20 minutes, but the headache got worse until she had to come to hospital. Her mouth twisted and felt tight. The doctors thought she’d had a stroke. While she was in the emergency department Simone tried to walk to the toilet but found she couldn’t move her legs. It felt almost like she had no legs. She couldn’t feel anything from the tops of her legs down. She had scans which were normal and the doctors said it probably wasn’t a stroke, but they didn’t know what had happened. The next day her stomach felt tight, and the nurses asked when she’d last passed urine. Simone realised she hadn’t been able to pass urine since before she came in. A bladder scan showed more than a litre. A catheter was put in. A scan of her spine and brain was done which were normal. Eventually a neurologist came to see Simone and found Hoover’s sign in her legs (weakness of hip flexion that normalises with contralateral hip extension). They explained that she had functional leg weakness triggered off by her first migraine and migraine aura. Simone did well with physiotherapy and found that within a few days she could walk again. Despite the legs getting better no one knew what was wrong with her bladder and it did not improve. |

**Careful history to identify predisposing and precipitating factors:** (medications, pain, urinary tract infections) and early removal of catheter if no clear aetiology for retention.

**Optimise first catheterisation experience:** A catheter was needed but using a lot of instillagel and giving it time to work, trying breathing exercises or distraction techniques and trying to ensure the patient feels safe will all make the initial catheterisation less unpleasant

**Early discussion of likely diagnosis** if no structural cause found. Early referral for pain management and exploration of other comorbidity. Consider MDT referral- physiotherapy, OT, psychology, psychiatry.

**Refer to specialist pelvic health physiotherapists**.
